# Supplementary material for: Iterative improvement in the automatic modular design of robot swarms
Source: PeerJ Comput Sci. 2020 Dec 7;6:e322. doi: 10.7717/peerj-cs.322 (PMC7924708; doi:10.7717/peerj-cs.322)
Supplement: Supplemental Information 3 [file peerj-cs-06-322-s003.zip › argos3/doc/api/standalone/a00483_source.html]

ARGoS: plugins/robots/foot-bot/simulator/pointmass3d\_footbot\_model.h Source File


- Main Page
- Related Pages
- Namespaces
- Classes
- Files

- File List
- File Members

# plugins/robots/foot-bot/simulator/pointmass3d\_footbot\_model.h

Go to the documentation of this file.

```
00001 
00007 #ifndef POINTMASS3D_FOOTBOT_H
00008 #define POINTMASS3D_FOOTBOT_H
00009 
00010 namespace argos {
00011    class CPointMass3DEngine;
00012    class CPointMass3DFootBotModel;
00013    class CFootBotEntity;
00014 }
00015 
00016 #include <argos3/plugins/simulator/physics_engines/pointmass3d/pointmass3d_model.h>
00017 #include <argos3/core/simulator/entity/embodied_entity.h>
00018 #include <argos3/plugins/robots/foot-bot/simulator/footbot_entity.h>
00019 
00020 namespace argos {
00021 
00022    class CPointMass3DFootBotModel : public CPointMass3DModel {
00023 
00024    public:
00025       
00026       CPointMass3DFootBotModel(CPointMass3DEngine& c_engine,
00027                                CFootBotEntity& c_footbot);
00028 
00029       virtual ~CPointMass3DFootBotModel() {}
00030       
00031       virtual void Reset();
00032 
00033       virtual void UpdateFromEntityStatus();
00034       virtual void Step();
00035 
00036       virtual void CalculateBoundingBox();
00037 
00038       virtual bool CheckIntersectionWithRay(Real& f_t_on_ray,
00039                                             const CRay3& c_ray) const;
00040 
00041       virtual void UpdateOriginAnchor(SAnchor& s_anchor);
00042 
00043    private:
00044 
00046       CWheeledEntity& m_cWheeledEntity;
00047 
00049       CRadians m_cYaw;
00050 
00052       Real m_fAngularVelocity;
00053 
00055       const Real* m_fCurrentWheelVelocity;
00056    };
00057 
00058 }
00059 
00060 #endif
```

---

Generated on 10 Jul 2018 for ARGoS by 
 1.6.1
